# ^66^Ga-PET-imaging of GRPR-expression in prostate cancer: production and characterization of [^66^Ga]Ga-NOTA-PEG_2_-RM26

**DOI:** 10.1038/s41598-021-82995-7

**Published:** 2021-02-11

**Authors:** Sara S. Rinne, Ayman Abouzayed, Katherine Gagnon, Vladimir Tolmachev, Anna Orlova

**Affiliations:** 1 Department of Medicinal Chemistry, Uppsala University, Uppsala, Sweden; 2 GEMS PET Systems, AB, Uppsala, Sweden; 3 Department of Immunology, Genetics and Pathology, Uppsala University, Uppsala, Sweden; 4 Centrum for Oncotheranostics, National Research Tomsk Polytechnic University, Tomsk, Russia; 5 Science for Life Laboratory, Uppsala University, Uppsala, Sweden

**Keywords:** Prostate cancer, Particle physics

## Abstract

Molecular imaging of the gastrin-releasing peptide receptor (GRPR) could improve patient management in prostate cancer. This study aimed to produce gallium-66 (T_½_ = 9.5 h) suitable for radiolabeling, and investigate the imaging properties of gallium-66 labeled GRPR-antagonist NOTA-PEG_2_-RM26 for later-time point PET-imaging of GRPR expression. Gallium-66 was cyclotron-produced using a liquid target, and enriched [^66^Zn]Zn(NO_3_)_2_. In vitro, [^66^Ga]Ga-NOTA-PEG_2_-RM26 was characterized in GRPR-expressing PC-3 prostate cancer cells. In vivo, specificity test and biodistribution studies were performed 3 h and 22 h pi in PC-3 xenografted mice. microPET/MR was performed 3 h and 22 h pi. Biodistribution of [^66^Ga]Ga-NOTA-PEG_2_-RM26 was compared with [^68^Ga]Ga-NOTA-PEG_2_-RM26 3 h pi. [^66^Ga]Ga-NOTA-PEG_2_-RM26 was successfully prepared with preserved binding specificity and high affinity towards GRPR. [^66^Ga]Ga-NOTA-PEG_2_-RM26 cleared rapidly from blood via kidneys. Tumor uptake was GRPR-specific and exceeded normal organ uptake. Normal tissue clearance was limited, resulting in no improvement of tumor-to-organ ratios with time. Tumors could be clearly visualized using microPET/MR. Gallium-66 was successfully produced and [^66^Ga]Ga-NOTA-PEG_2_-RM26 was able to clearly visualize GRPR-expression both shortly after injection and on the next day using PET. However, delayed imaging did not improve contrast for Ga-labeled NOTA-PEG_2_-RM26.

## Introduction

The gastrin-releasing peptide receptor (GRPR) is a receptor of the bombesin family and is overexpressed in 63–100% of primary prostate cancers^1–5^. Expression of GRPR in prostate cancer is heterogeneous, dynamic and dependent on the stage of the disease^1,2,6^. Overexpression of GRPR and GPRR-mediated signaling can stimulate the growth of both androgen-dependent and androgen-independent prostate cancer cells^7,8^, indirectly promote angiogenesis^9^, and increase the invasive potential of prostate cancer^10,11^. GRPR could be used for imaging of prostate cancer spread due to its expression pattern. Early detection of dissemination and characterization of prostate cancer is vital, because the treatment regimen is dependent on the stage and molecular characteristics of the disease. Frequently used diagnostic methods, for both primary and recurrent prostate cancer, include screening for elevated concentration of the prostate specific antigen (PSA) in blood and biopsy sampling, along with digital rectal exam, and anatomical imaging. However, these methods have limited sensitivity. Elevated PSA-levels are not prostate cancer specific^12^, and efficiency and practicality of biopsy sampling is restricted by tumor heterogeneity, metastasized disease and the invasive procedure^13^.

Radionuclide-based molecular imaging is a non-invasive tool of increasing relevance for the detection of molecular targets, patient stratification and treatment follow-up in prostate cancer and other diseases. Positron emission tomography (PET) and single photon emission tomography (SPECT) can complement the existing methods for the detection of primary and recurrent prostate cancer, and provide whole-body information about the molecular disease characteristics. Additionally, they could be used for planning of surgical intervention, external beam therapy, targeted radiotherapy, and therapy monitoring. There are indications that GRPR is mainly overexpressed in earlier stages of prostate cancer^1,2,6^, while healthy prostate tissue remains GRPR-negative^2^. In addition, expression was also documented in metastases located for examples in lymph nodes and bones^14^. This further strengthens the potential of GRPR as a target for PET and SPECT-imaging in prostate cancer, particularly at the oligometastatic stage.

Bombesin is a 14 amino acid peptide that shares the same seven amino acid sequence at C-terminus with the gastrin-releasing peptide (natural ligand for GRPR). Bombesin can bind GRPR with high affinity, and in recent years, a multitude of bombesin-derived radioligands have been developed for PET- and SPECT-imaging and GRPR-targeted therapy^15–18^. The first generation of GRPR-targeting peptides mainly included the development of receptor agonists, which were thought to be favorable because they would provide quick internalization and good retention of the radiotracer. However, the undesirable side effects in the GI-tract upon binding and internalization of GRPR-agonists, and the success reported for somatostatin antagonists^19^ resulted in a shift of this paradigm, favoring the development of GRPR-antagonists^20^.

Our group has extensively investigated the GRPR-antagonist RM26 (D-Phe–Gln–Trp–Ala–Val–Gly–His–Sta–Leu–NH_2_) for SPECT- and PET-imaging, and targeted therapy^21–25^. Due to the high binding-affinity, excellent uptake of RM26 in GRPR-expressing tumors, the low accumulation in non-expressing organs, and the quick clearance from circulation ^68^Ga-, ^55/57^Co- and ^111^In-labeled RM26 variants were able to clearly visualize GRPR-expression in preclinical models^21,22,24^. Recently, a clinical study including 28 individuals (healthy volunteers, patients with newly diagnosed PC, and post-therapy patients) showed the safety and feasibility of [^68^Ga]Ga-RM26 for imaging of primary GRPR-expressing primary tumors, as well as lymph node and bone metastases^26^.

High PET and SPECT image contrast is important for the diagnostic accuracy, especially for small lesions. Our experience with ^55/57^Co and ^111^In-labeled RM26 showed that the imaging contrast improved with later time points, due to the continued clearance of the tracer from normal organs^21,22^. PET-imaging generally has higher sensitivity than SPECT^27^. Unfortunately, there is a noticeable shortage of longer-lived radionuclides suitable for delayed PET-imaging. Clinically used nuclides ^68^Ga and ^18^F with half-lives of 68 min and 110 min, respectively, only allow for imaging up to few hours after injection. ^89^Zr is used for immuno-PET imaging, but the half-life of 3.27 days is rather unfavorable for targeting agents with fast pharmacokinetics. PET-radionuclides with intermediate half-lives (several hours up to a day) could be an ideal match for smaller imaging agents such as short peptides or engineered scaffold proteins (ESPs), because they expand the imaging time-window, while lowering the radiation dose burden to the patient. Positron emitting isotopes ^64^Cu (T_1/2_ = 12.7 h) and ^86^Y (T_1/2_ = 14.7 h) have been explored for PET-imaging, but have a relatively low positron-abundance (17.8% and 34% respectively)^28^; ^55^Co (T_1/2_ = 17.5 h) and ^44^Sc (T_1/2_ = 4 h), have more desirable properties, however the half-life of ^44^Sc could be a limiting parameter for next-day imaging.

The gallium-isotope ^66^Ga is another potential alternative to the scarcity of longer-lived positron-emitting radionuclides. Gallium-66 has a half-life of 9.5 h, a positron abundance of 56.5%^29^, and can be cyclotron-produced by irradiation of natural or enriched ^66^Zn-targets^30^. One potential drawback however is the high energy of the emitted positrons (up to 4.15 MeV), because it could impact spatial resolution^31,32^. Regardless, promising studies have been published evaluating ^66^Ga for PET-imaging of tumor angiogenesis using a CD105-targeting antibody^33^, as well as imaging of α_v_β_3_^34^, PSMA^35^ and somatostatin receptor^36^ with ^66^Ga -labeled peptides. PET-images could be acquired as late as 36 h^33^ and 48 h post injection (pi)^35^. The longer half-life, possible cyclotron-production and the fact that gallium-chemistry is already established for many PET-tracers, make it an interesting nuclide for delayed PET-imaging.

One aim of the present study was to apply existing technology developed for direct cyclotron-based production of ^68^Ga using a liquid target (i.e. by irradiation of an isotopically enriched salt solution of ^68^Zn) and chemical processing to isolate [^68^Ga]GaCl_3_to the production of ^66^Ga and [^66^Ga]GaCl_3._ The second aim of this study was to radiolabel and investigate the imaging properties of ^66^Ga for PET-imaging of GRPR expression over time using the bombesin-like peptide NOTA-PEG_2_-RM26 in a preclinical prostate cancer model. In addition, we included ^68^Ga-labeled NOTA-PEG_2_-RM26 for early time point comparison.

## Results

### ^66^Ga production and purification

^66^Ga was successfully produced in a liquid target with end-of-bombardment (EOB) yields up to 0.50 GBq, thus corresponding to saturation yields of 0.25 GBq/μA. When combining all fractions, the isolated [^66^Ga]GaCl_3_ activity was ~ 320 to 340 MBq, of which the fractions used for radiolabeling had activity concentrations ranging from 160 to 280 MBq/mL.

A coarse spot check was performed on one [^66^Ga]GaCl_3_ production of which Zn content in the two fractions preceding, and two fractions following the fraction used for radiolabeling were all below the lowest positive color scale of 4 μg/mL. Isolated fractions of [^66^Ga]GaCl_3_ were not analyzed for residual Zn due to the small fraction volumes. However, extensive tests have been performed previously during ^68^Ga development efforts, whereby, residual Zn was determined to be 0.33 ± 0.23 μg/mL.

The gamma-spectrum of ^66^Ga is presented in Figure S1. The only observable gamma-lines were 511, 834 and 1039 keV belonging to ^66^Ga. No other gamma-lines were observed. Data concerning half-live measurement of ^66^Ga are presented in Figure S2. The measured data were perfectly fitted (R^2^ = 1) in a monoexponetial decay with a the half-life of 9.45 ± 0.05 h, which is in an excellent agreement with the half-life of ^66^Ga (9.49 h, http://nucleardata.nuclear.lu.se/toi/nucSearch.asp ; The Lund/LBNL Nuclear Data Search).

Taken together, the data confirm authenticity and high radionuclide purity of ^66^Ga.

### Labeling

Labeling of NOTA-PEG_2_-RM26 with ^66^Ga resulted in 99% ± 1% (n = 7) radiochemical yield determined by ITLC. Specific activity was in the range of 2.5–5 GBq/mg (molar activity if 3.9–7.8 MBq/nmol) and no significant release of ^66^Ga was observed after incubation in 1000-fold molar excess of EDTA, PBS or human serum for 1 h (numerical available in supplementary Table S1). Because of the almost quantitative radiochemical yield, [^66^Ga]Ga-NOTA-PEG_2_-RM26 was used without further purification.

**Figure 1:**
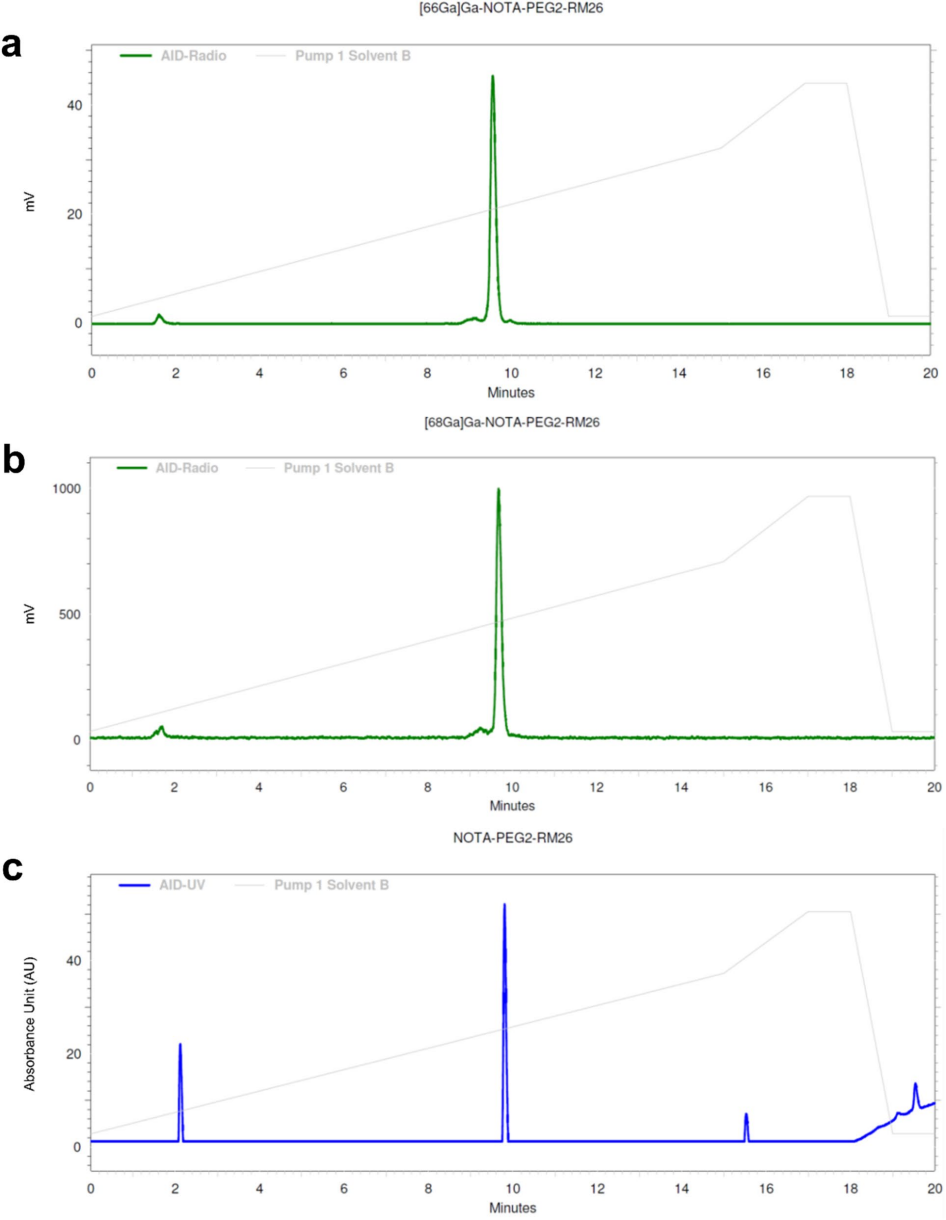
HPLC chromatograms of **(a)** [^66^Ga]Ga-NOTA-PEG_2_-RM26, **(b)** [^68^Ga]Ga-NOTA-PEG_2_-RM26 and **(c)** NOTA-PEG_2_-RM26. HPLC analysis was done using a C18 column and a gradient of 5 to 70% acetonitrile (with 0.1% TFA) in water over 15 min.

Radio-HPLC analysis demonstrated identity of [^66^Ga]Ga-NOTA-PEG_2_-RM26 (Fig. 1a,c). There were no differences in HPLC profiles of [^66^Ga]Ga-NOTA-PEG_2_-RM26 and [^68^Ga]Ga-NOTA-PEG_2_-RM26 (Fig. 1a,b).

### Binding specificity, cellular processing and analysis of binding kinetics

**Figure 2:**
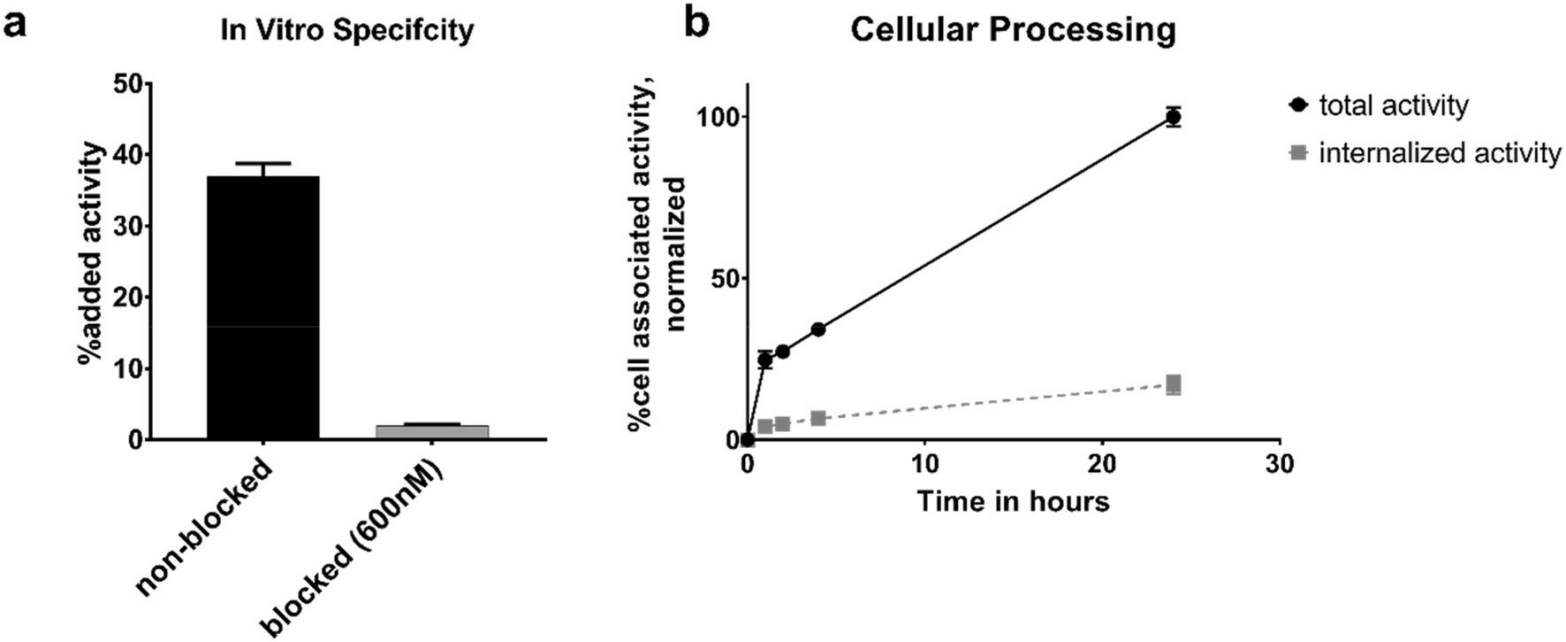
In vitro characterization of [^66^Ga]Ga-NOTA-PEG_2_-RM26 on GRPr expressing PC-3 cells. **(a)** In vitro binding specificity. Receptors in the blocked group were pre-saturated with 600 nM NOTA-PEG_4_-RM26 before incubation with 1 nM [^66^Ga]Ga-NOTA-PEG_2_-RM26 for 1 h at 37 °C. **(b)** Cellular Processing. Cells were continuously incubated with [^66^Ga]Ga-NOTA-PEG_2_-RM26 for up to 24 h at pre-selected time points membrane bound and internalized activity were collected. All data is displayed as average ± standard deviation (n = 3).

Blocking of GRPR in both cell lines cells resulted in significantly (p < 0.008) lower cell-associated activity with both concentrations of blocking peptide (Fig. 2a, Figure S3) compared with the non-blocked cells. Uptake in DU145 cells was significantly lower than in PC-3 cells. Binding of [^66^Ga]Ga-NOTA-PEG_2_-RM26 to cells was rapid and the amount of total cell-associated activity increased constantly with time. Internalized fraction slowly, but steadily increased over 24 h. The fraction of internalized activity was 17% of cell-associated activity at the end of the observation period (Fig. 2b).

**Figure 3:**
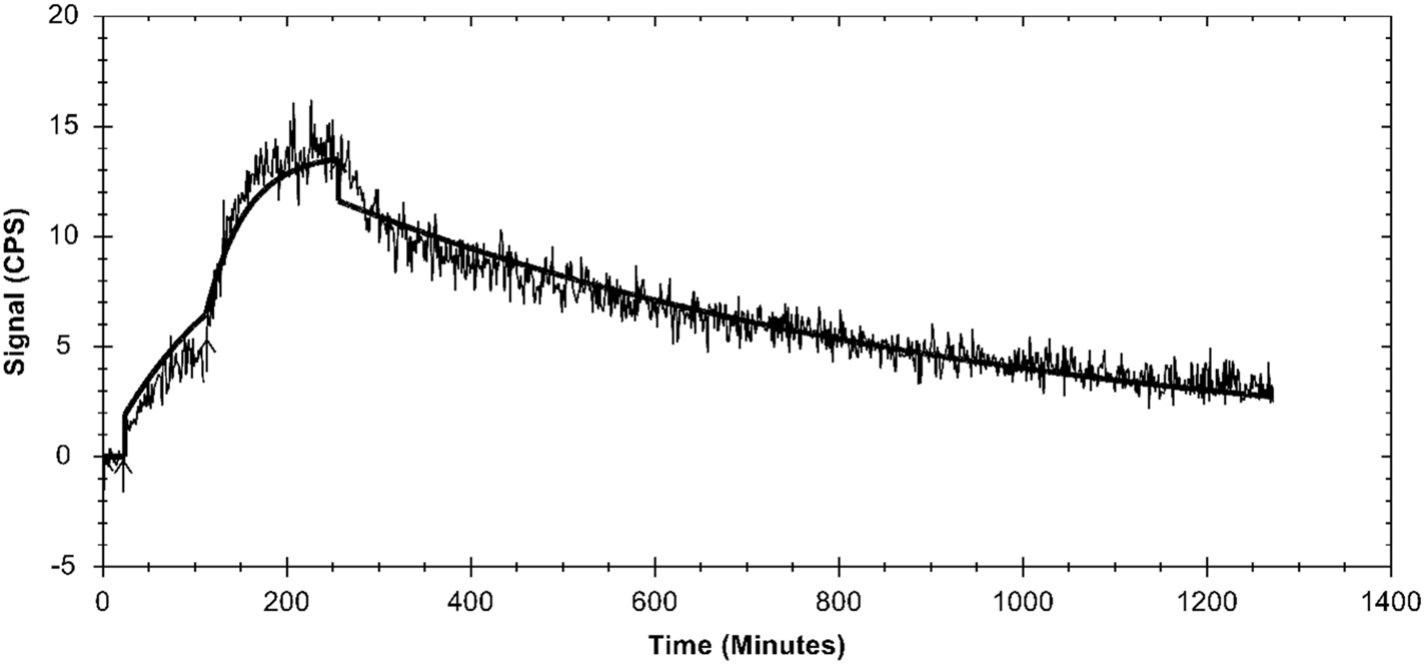
Representative curve from real-time Ligand Tracer measurements of binding kinetics of [^66^Ga]Ga-NOTA-PEG_2_-RM26. GRPR-expressing PC-3 cells were plated in a dedicated area of 10 cm petri dish an and increasing concentrations of [^66^Ga]Ga-NOTA-PEG2-RM26 were added stepwise to record association. Solution was replaced with fresh culture media to record dissociation. Data were analyzed with a 1:1 kinetic binding model using TraceDrawer Software (Ridgeview Instruments AB).

Binding kinetics were measured in real-time using LigandTracer and data were fitted using a 1:1 kinetic binding model. A representative Ligand Tracer curve is displayed in Fig. 3. Equilibrium binding constant (K_D_) was 189 ± 50 pM with an association rate (k_a_) of 1.78 × 10^5^ ± 0.05 × 10^5^ 1/Ms and a dissocation rate (k_d_) of 3.3 × 10^–5^ ± 0.8 × 10^–5^ 1/s.

### Biodistribution and in vivo specificity

**Figure 4:**
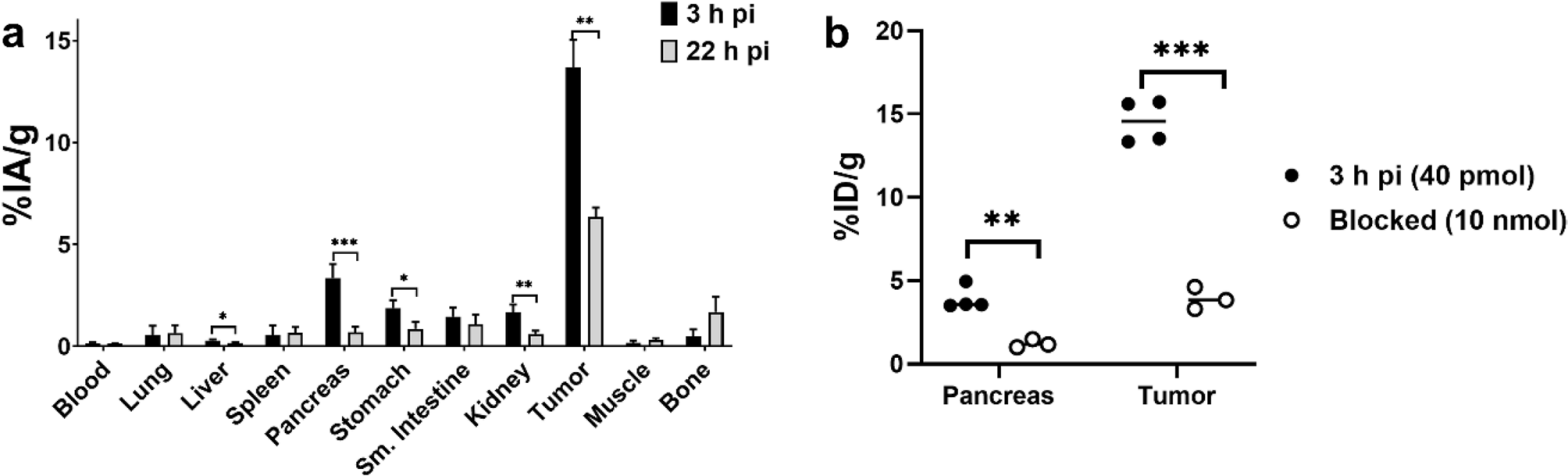
**(a)** Biodistribution and **(b)** in vivo specificity of [^66^Ga]Ga-NOTA-PEG_2_-RM26. Female balb/c nu/nu mice with PC-3 xenografts were injected with 40 pmol (40 kBq) [^66^Ga]Ga-NOTA-PEG_2_-RM26. For the in vivo specificity test mice in the blocked group were co-injected with 10 nmol non-labeled NOTA-PEG_2_-RM26. Data is presented as %IA/g as average ± standard deviation (n = 3–4 animals/group). Data for GI and body is expressed as %IA. * indicates statistical significant difference (p < 0.05).

Biodistribution of [^66^Ga]Ga-NOTA-PEG_2_-RM26 was studied 3 h and 22 h pi in Balb/c nu/nu mice bearing PC-3 xenografts. [^66^Ga]Ga-NOTA-PEG_2_-RM26 cleared rapidly from blood and bound to GRPR-expressing xenografts as well as organs with natural expression of (murine) GRPR (pancreas, stomach, small intestine) (Fig. 4a). In vivo specificity test showed significant decrease in uptake in GRPR expressing xenografts (p < 0.00001) and pancreas (p < 0.002) indicating GRPR specific binding / of [^66^Ga]Ga-NOTA-PEG_2_-RM26 (Fig. 4b). Tumor uptake 3 h pi was 14 ± 1% ID g but decreased almost two-fold 22 h pi. However, the tumor uptake was higher than the uptake in all normal organs at both time points. Uptake in GRPR-expressing pancreas decreased significantly (p < 0.004) from 3 to 22 h pi, as well as in GI and carcass. Noticeably, the uptake in bone increased during the observation period. No increase in tumor-to-organs ratios was observed form 3 to 22 h pi (Table 1). Tumor-to-organ ratios were generally higher 3 h pi for all organs except pancreas.

**Table 1:** Tumor-to-organ ratios.

	3 h pi	22 h pi
Blood	57 ± 10*	28 ± 4
Lung	11 ± 2*	5 ± 1
Liver	40 ± 5*	28 ± 3
Spleen	11 ± 3*	5 ± 1
Pancreas	3.8 ± 0.5*	5.0 ± 0.9
Stomach	6 ± 1	4.1 ± 1.0
Sm. intestine	6 ± 2	3 ± 1
Kidney	7.3 ± 1.0	7 ± 1
Muscle	39 ± 13*	11 ± 2
Bone	10 ± 3*	2 ± 1

Female balb/c nu/nu mice with PC-3 xenografts were injected with 40 pmol (40 kBq) ^66^Ga-NOTA-PEG_2_-RM26.

*Indicates statistically significant (p < 0.03) differences between groups.

Comparison of [^66^Ga]Ga-NOTA-PEG_2_-RM26 with its [^68^Ga]Ga-labeled counterpart showed no remarkable differences in uptake in studied organs and tissues (Table 2).

**Table 2:** Head-to-head comparison of [^66^Ga]Ga-NOTA-PEG_2_-RM26 and [^68^Ga]Ga-NOTA-PEG_2_-RM26 3 h pi in female Balb/c nu/nu mice.

	^66^Ga	^68^Ga
Blood	0.15 ± 0.06	0.24 ± 0.05
Lung	0.5 ± 0.4	0.26 ± 0.08
Liver	0.28 ± 0.06	0.30 ± 0.05
Spleen	0.6 ± 0.4	0.21 ± 0.04
Pancreas	3.4 ± 0.7	3.3 ± 0.9
Stomach	1.9 ± 0.4	1.8 ± 0.3
Sm. Intestine	1.4 ± 0.4*	0.6 ± 0.2
Kidney	1.7 ± 0.4	3 ± 1
Tumor	14 ± 1	14 ± 5
Muscle	0.2 ± 0.1	0.07 ± 0.03
Bone	0.5 ± 0.3	0.3 ± 0.1
GI	2.9 ± 0.3	3.0 ± 0.7
Body	1.3 ± 0.2*	3 ± 1

Data is presented as %ID/g (average ± standard deviation, n = 3–4).

*Indicates statistically significant (p < 0.01) differences between groups.

Distribution of free gallium-66 in NRMI mice 3 h pi (Figure S4) showed slow excretion of activity with relatively equal activity distribution in healthy organs. Radio-HPLC analyses of blood plasma demonstrated that 5 min pi of [^68^Ga]Ga-NOTA-PEG_2_-RM26 up to 57% of radiometal is associated with intact peptide (Figure S5). Free gallium-66 represented 6% of activity in blood plasma, while the rest of activity was associated with different peptide’s fragments.

### microPET/MR imaging

**Figure 5:**
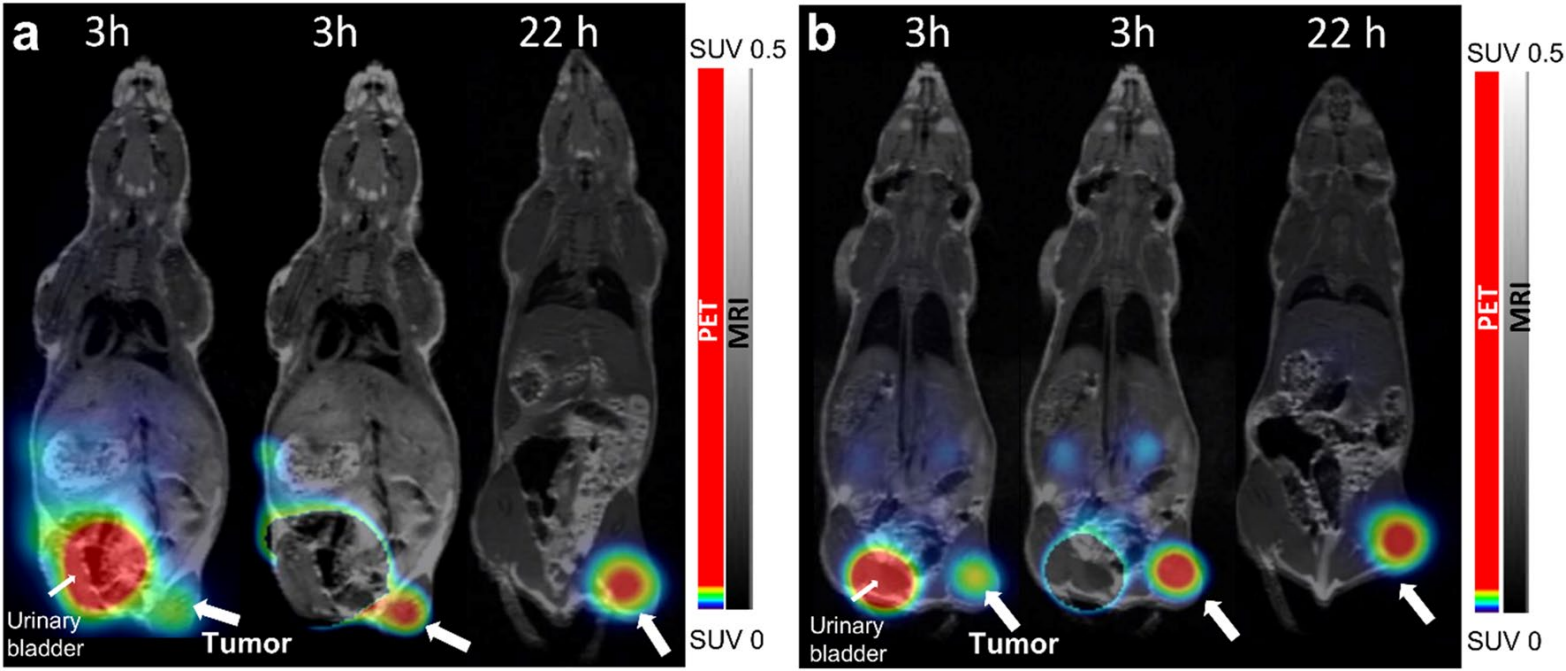
microPET/MR scans of PC-3 xenograft bearing mice at 3 h and 22 h pi. **(a,b)** show scans of two different animals injected intravenously with 40 pmol (0.25 MBq) [^66^Ga]Ga-NOTA-PEG_2_-RM26 each. Mice were anaesthetized during the 3 h scan, and sacrificed before the 22 h pi scan. 3 h pi scans are displayed with a signal from urinary bladder (left) and with the signal from urinary bladder removed (middle) after reconstruction.

microPET/MR imaging was performed 3 h and 22 h pi and images are displayed in Fig. 5. GRPR-expressing xenografts could be clearly visualized at both time points. Tumors showed the highest uptake aside from urinary bladder 3 h pi.

## Discussion

Molecular imaging in prostate cancer could improve the diagnostic accuracy for both primary and recurrent disease and thus improve patient management. The GRP receptor is overexpressed in mainly earlier stages of prostate cancer, but not in healthy prostate tissue^1,2^, and is thus an attractive target for diagnostic PET- and SPECT- imaging. The GRPR-antagonist PEG_2_-RM26 (PEG_2_-DPhe-Gln-Trp-Ala-Val-Gly-His-Sta-Leu-NH_2_) is a promising ligand for imaging of GRPR-expression^18^. First steps towards clinical translation using ^68^Ga-labeled RM26 have been reported recently assessing the safety, biodistribution, and dosimetry in humans^26^. Many ^68^Ga-labeled tracers are successfully used in clinical routine for imaging shortly after injection. Pre-clinical studies by our group suggested that imaging contrast, and thereby sensitivity, could be improved by next-day imaging^21,22^. However, limited information is available about the long-term fate of gallium-labeled probes and their potential for later time-point imaging, due to the limited half-life of ^68^Ga. Gallium-66 is another positron-emitting gallium-isotope, which, because of its longer half-life of 9.5 h, could be an interesting addition to the PET-toolbox for later-time point imaging. The aims of the present study were to produce ^66^Ga by cyclotron irradiation of enriched ^66^Zn- in a liquid target and to investigate the PET-imaging properties of [^66^Ga]Ga-NOTA-PEG2-RM26 for later time point imaging of GRPR-expression.

Although the ^68^Ga used in this study was acquired from a ^68^Ge/^68^Ga generator, this study demonstrated that the alternative technology developed for the direct cyclotron-based production of ^68^Ga with a liquid target could be readily adopted to production of ^66^Ga by irradiation of a ^66^Zn salt solution, with the [^66^Ga]GaCl_3_ of suitable quality for radiolabeling. Although yields afforded by a liquid target are significantly less than those which can be obtained using a solid target, the achieved yields of ^66^Ga were nevertheless adequate to enable in vitro and in vivo pre-clinical studies without the need for sophisticated solid-target infrastructure.

The GRPR-antagonist NOTA-PEG_2_-RM26 was labeled with ^66^Ga with yield similar to [^68^Ga]Ga-PEG_2_-RM26^24,37^. Also, the in vitro and in vivo stability tests did indicate good stability of the ^66^Ga-NOTA complex. Because isotopes have identical chemical properties, this was expected. In contrast to the cyclotron-produced ^66^Ga, ^68^Ga is typically eluted from a ^68^Ge/^68^Ga-generator (as was the case for this study) and the different production routes may potentially result in different impurity profiles of the radioisotope solutions, which in return could affect the radiolabeling efficiency. However, such differences were not observed in this study—this was also confirmed by HPLC analysis of both radiolabeled products.

As expected, the GRPR binding specificity of [^66^Ga]Ga-NOTA-PEG_2_-RM26 was retained after labeling and the difference in uptake for PC-3 and DU145 cells correlated well to the different levels of receptor expression^38^. For the first time, binding affinity of gallium-labeled NOTA-PEG_2_-RM26 towards GRPR was measured directly and in real-time showing a K_D_ value in the low picomolar range similar to the earlier reported K_D_ for [^111^In]In-NOTA-PEG_2_-RM26^21^. Due to the longer half-life of ^66^Ga, we were also able to follow the cellular processing of gallium-labeled NOTA-PEG_2_-RM26 past 4 h. The cellular processing pattern of [^66^Ga]Ga-NOTA-PEG_2_-RM26 at early time points matched published data for [^68^Ga]Ga-NOTA-PEG_2_-RM26^37^. Beyond the 4 h the internalized fraction of [^66^Ga]Ga-NOTA-PEG_2_-RM26 remained low, with only 17% of the total cell associated activity internalized after 24 h. Slow internalization was expected due to the antagonistic properties of RM26. However, the low level of internalized activity resembled the behavior of radiocobalt-labeled NOTA-PEG_2_-RM26^22,39^, and was considerably lower than the level of internalization of its [^111^In]In-labeled analog^21^. Similar to [^55/57^Co]Co-PEG_2_-RM26, the radiocobalt label also led to lower amounts of internalized activity with affibody molecules, and it is hypothesized that the radiocobalt label leaks from or is transported out of the cell by specific cobalt-efflux mechanisms^22,39–41^. It could be speculated that similar mechanisms are in place for gallium.

The general biodistribution pattern of [^66^Ga]Ga-NOTA-PEG_2_-RM26 was comparable with previous studies of RM26 labeled with different radiometals^21,22,24^. [^66^Ga]Ga-NOTA-PEG_2_-RM26 cleared rapidly from blood mainly via the renal pathway, and specific uptake of [^66^Ga]Ga-NOTA-PEG_2_-RM26 was observed in GRPR-positive pancreas and the PC-3 xenografts. The release of activity from tumors with time, that was observed for [^66^Ga]Ga-NOTA-PEG_2_-RM26 was also observed for [^111^In]In- and radiocobalt-labeled NOTA-PEG_2_-RM26. However, the type of radiometal used for radiolabeling appears to influence the retention of activity in the tumor. Among the ^66^Ga-, radiocobalt- and ^111^In-labeled tracers, ^111^In had the best retention in tumors with more than 65% of initial tumor-associated activity remaining 24 h pi^21^. [^55/57^Co]Co-NOTA-PEG_2_-RM26 had the fastest release from tumors with less than 30% of activity remaining 24 h pi^22,39^, while in the case [^66^Ga]Ga-NOTA-PEG_2_-RM26 approximately 50% of activity was retained in tumors from 3 to 22 h pi. It is possible that the different coordination geometries of the [^111^In]In-NOTA complex compared to the gallium- and cobalt-NOTA complex can have an effect on the biodistribution and tumor retention^18,42,43^. Regardless, the retention pattern of these RM26-variants in vivo correlated well with the internalization in the in vitro assays and the suspected leakage of radiocobalt and gallium-catabolites form cells. This suspicion might be further supported by data from Heppeler et al.^44^ whereby, similar to our study, [^111^In]In-DOTA-TOC had improved tumor retention compared with [^67^Ga]Ga-DOTA-TOC from 4 to 24 h pi.

As seen previously with the [^111^In]In- and [^55/57^Co]Co-labeled NOTA-PEG_2_-RM26, [^66^Ga]Ga-NOTA-PEG_2_-RM26 cleared from the GRPR positive pancreas more rapidly than from the tumor, which could be attributed to the differences between human and murine GRPR as well as to the different receptor densities in tumors compared to pancreas. Analyses of blood plasma demonstrated that gallium labeled NOTA-PEG_2_-RM26 has metabolic stability similar to other GRPR-targeting peptides; 5 min pi up to 60% of radiometal is associated with peptide. For comparison, when injected without neprilysin -inhibitor phosphoramidon only 25–30% of activity was associated with truncated human endogenous GRP motifs^45^, for [^111^In]In‐SB9 and [^177^Lu]Lu-PEG2-RM26 65–80% of injected peptide were intact at this time^23,46^. Analyses of blood plasma also demonstrated that [^66^Ga]Ga-NOTA complex was preserved under metabolic degradation because free radiometal represented only small part of all activity in blood. Interestingly, [^66^Ga]Ga-NOTA-PEG_2_-RM26 did not clear as efficiently from other organs. This might further support the speculation that gallium leaks from cells after receptor-mediated internalization. The redistribution of radiocatabolites could be a possible explanation for the elevated uptake of [^66^Ga]Ga-NOTA-PEG_2_-RM26 in bone, since we observed good in vitro stability of [^66^Ga]Ga-NOTA-PEG_2_-RM26. Unfortunately, because of the limited clearance of [^66^Ga]Ga-NOTA-PEG_2_-RM26 from healthy tissues, tumor-to-organ ratios did not improve from 3 to 22 h pi. Biodistribution of [^68^Ga]Ga-NOTA-PEG_2_-RM26 was in agreement with biodistribution of [^66^Ga]Ga-NOTA-PEG_2_-RM26 and data published earlier^24,37^.

The microPET/MR images showed excellent visualization of the GRPR-expressing xenografts. For imaging of prostate cancer high contrast in the abdominal region is essential. Despite the somewhat unexpected biodistribution of [^66^Ga]Ga-NOTA-PEG_2_-RM26 without increase in tumor-to-organ ratios over time, the imaging contrast remained excellent even at 22 h pi due to clearance from GI-tract and whole body with time. Gallium-66 has a rather high positron branching ratio (i.e. 57%) compared with other positron emitters for PET imaging with intermediate (^64^Cu, 18% β^+^; ^86^Y, 32% β^+^) or longer (^89^Zr, 23% β^+^) half-lives. However, the maximum energy of positron emitted from ^66^Ga (4.15 meV) is higher than for many other positron-emitters, and could affect image quality. Nevertheless, the results from our study and others investigating ^66^Ga -labeled peptides and monoclonal antibodies for PET-imaging of other molecular targets, such as somatostatin receptor^36^, α_V_β_3_^34^_,_ and CD105^33^ underline the potential of ^66^Ga as a valuable addition to the PET-imaging toolbox.

## Conclusion

In conclusion, we successfully produced ^66^Ga by cyclotron irradiation of a liquid [^66^Zn]Zn(NO_3_)_2_ target, followed by subsequent purification, and radiolabeled the bombesin antagonist NOTA-PEG_2_-RM26 with this radionuclide. We further demonstrated the feasibility of [^66^Ga]Ga-NOTA-PEG_2_-RM26 for PET-imaging of GRPR expression. In contrast to our initial hypothesis, early and late time point imaging provided similar image quality in our case. Nevertheless, prolonged half-life, and the widely explored gallium-chemistry could make ^66^Ga an attractive radionuclide for PET-imaging with other targeting agents that have slightly extended biological half-lives such as engineered scaffold proteins.

## Material and methods

### General

The bombesin-analog NOTA-PEG_2_-RM26 (NOTA-PEG_2_-DPhe–Gln–Trp–Ala–Val–Gly–His–Sta–Leu–NH_2_) was produced by Pepmic Co., Ltd. (Suzhou High-tech Development Zone, Suzhou, Jiangsu, China 215151) upon our order. The GRPR-expressing prostate cancer cell line PC-3 was purchased from American Type Tissue Collection (ATCC via LGC Standards AB, Borås, Sweden) and cultured in RMPI 1640 cell culture media (Sigma Aldrich, St. Louis, MO, United States) supplemented with 20% fetal bovine serum (FBS, Sigma Aldrich, St. Louis, Missouri, United States), 1% penicillin–streptomycin and 1% l-glutamine (both Biochrom, Berlin, Germany). Trypsin–EDTA (Sigma Aldrich, St. Louis, MO, United States) was used to detach cells.

Animal experiments were approved by the Ethics Committee for Animal Research in Uppsala, Sweden and performed according to the Swedish national legislation on protection of laboratory for animals and carried out in compliance with the ARRIVE guidelines. Female Balb/c nu/nu mice were obtained from Scanbur A/S (Karlslunde, Denmarks and house at 22 °C, 48% humidity, 12/12 h light/dark cycle. Standard laboratory food and water were available ad libitum. Mice with GRPR-expressing PC-3 xenografts (implanted 21 d before the experiment) were used for all in vivo studies. Groups of 3–4 mice were used per data point.

^68^Ga was obtained by elution of an ^68^Ge/^68^Ga generator (Cyclotron Co. Obninsk, Russia) with metal free 0.1 M HCl. An automated gamma counter with a 3-inch NaI(Tl) detector (2480 Wizard; Wallac Oy, Turku, Finland) was used to measure the activity content in cell and tissue samples.

Statistical significance (p-value < 0.05) was determined with unpaired, two tailed t-test using GraphPad Prism software (version 7.03 for Windows, GraphPad Software Inc., San Diego, CA, United States).

### Cyclotron-based ^66^Ga production and characterization of ^66^Ga

The ^66^Ga was produced by leveraging the GE PETtrace ^68^Ga liquid target and GE FASTlab [^68^Ga]GaCl_3_ purification chemistry. The technology is described in further detail in^47,48^ with an additional intermediate strong anion exchange resin introduced early 2019 to further reduce trace metal impurities for sites exploring [^68^Ga]Ga-DOTA-TOC and [^68^Ga]Ga-DOTA-TATE labelling applications.

Namely, a solution of 1.0 M isotopically enriched [^66^Zn]Zn(NO_3_)_2_ in excess 0.3 M HNO_3_ was prepared from enriched [^66^Zn]ZnO (Isoflex, ^64^Zn: 0.03%; ^66^Zn: 99.07%; ^67^Zn: 0.70%; ^68^Zn: 0.18%; ^70^Zn: 0.01%), HNO_3_ (70%; ≥ 99.999% trace metal basis; Sigma-Aldrich), and MilliQ 18 MΩ-cm water. The solution (2.2 mL per irradiation) was irradiated at a nominal proton energy of 14.3 MeV, for 70–75 min at approximately 25 μA and transferred through a capillary line to an external collection vial which was connected to the FASTlab.

**Figure 6:**
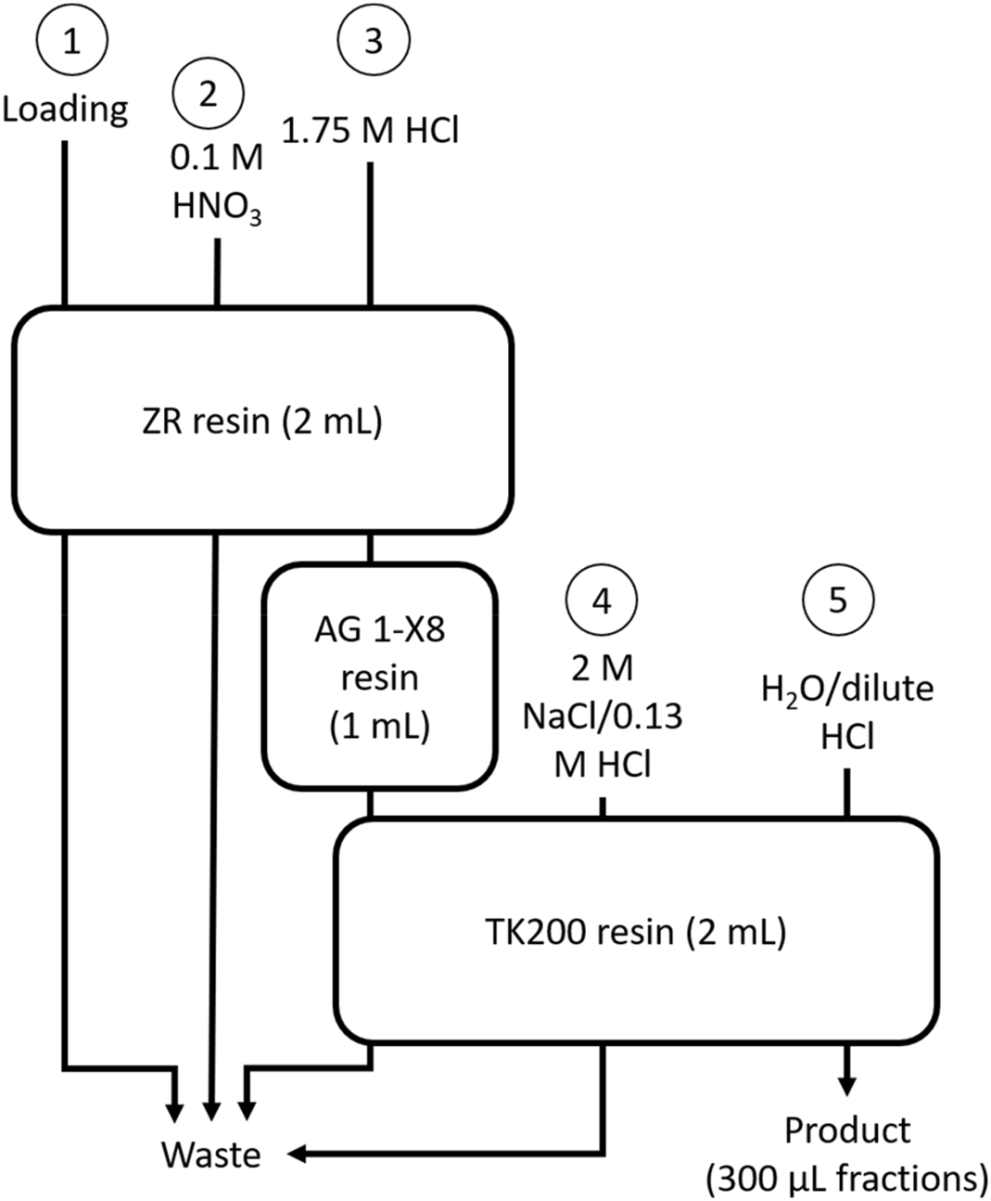
Three column approach for isolating [^66^Ga]GaCl_3_. Process steps described below. **(1)** The diluted solution was loaded at a rate of ~ 1.5 mL/min over a 2 mL ZR column, whereby ^66^Ga is retained on the resin, and the irradiated ^66^Zn was passed through to a collection vial for optional recycling. During the irradiation, coproduced ^13^N is passed through in this step as well. **(2)** The ZR resin was rinsed with 14 mL of 0.1 M HNO_3_ to remove residual ^66^Zn. **(3)** The ^66^Ga was eluted from the ZR resin with 5 mL 1.75 M HNO_3_, passed over a 1 mL AG 1-X8 column to remove residual trace metals, before being trapped on a TK200 column. **(4)** The TK200 column was rinsed with 3.5 mL 2 M NaCl in 0.13 M HCl to decrease residual acid on the column. **(5)** Finally, the ^66^Ga, as [^66^Ga]GaCl_3_ was eluted from the TK200 resin in nominal 300 μL fractions, the first 3.5 mL of which was water, followed by 2.5 mL 0.18 M HCl. The fraction(s) with the highest radioactivity concentration(s) were used for radiolabeling.

The irradiated solution was subsequently diluted in the collection vial with water to a total volume of ~ 8 to 9 mL, and automatically processed on a FASTlab Developer platform as outlined and described in Fig. 6. All resins noted below were obtained from Triskem (pre-packed, Britany, France), with additional reagents obtained as follows: HCl (30%; Ultrapure; Merck), HNO_3_ (70%; ≥ 99.999% trace metal basis; Sigma-Aldrich), NaCl (99.999% trace metal basis; Sigma Aldrich).

A coarse spot check was performed on one [^66^Ga]GaCl_3_ production using semi-quantitative colorimetric test strips (Merck, MQuant), of which Zn content in the two fractions preceding, and two fractions following the fraction used for radiolabeling. Isolated fractions of [^66^Ga]GaCl_3_ were not analyzed via ICP-MS (inductively coupled plasma mass spectrometry) for residual Zn due to the small fraction volumes, but extensive tests have been performed previously during ^68^Ga development efforts, whereby an identical separation scheme was used (albeit a non-fractionated 5 mL product volume (n = 12)).

To confirm authenticity and radionuclide purity of ^66^Ga, the half-life and gamma-spectra of the product were measured. The gamma spectra were measured using ultra-pure germanium detector (Mirion Technologies, San Ramon, CA, US) working in line with the DSA-LX multi-channel analyser (Mirion Technologies, San Ramon, CA, US). Analysis was performed using Genie 2000 software (Mirion Technologies). To determine the half-life, two samples were repeatedly measured using 1480 Wizard gamma-spectrometer during 70 h. Count rate was measured in the range from 10 to 2048 keV. Data fitting was performed using GraphPad software.

### Labeling of NOTA-PEG_2_-RM26, and in vitro stability and HPLC analysis of [^66^Ga]Ga-NOTA-PEG_2_-RM26

For labeling of NOTA-PEG_2_-RM26 with ^66^Ga, 3–10 nmol of peptide was buffered with 1.25 M sodium acetate buffer, pH 3.6, and incubated with 4–8 MBq/nmol for 12 min at 85 °C. Radiochemical yield was determined by instant thin-layered chromatography (ITLC). For this, a sample of the reaction mixture was applied to silica gel-impregnated glass microfiber chromatography paper (Agilent Technologies, Santa Clara, CA, USA), which was eluted with 0.2 M citric acid. Free gallium moves with the citric acid to the front of the chromatography paper, whereas the radiolabeled peptide remains at the application point. The activity distribution was measured using ScanRam radio-TLC Scanner and analyzed with complimentary Software, Laura (v6.0.4.92) (LabLogic, Sheffield S10 2QJ, UK) To test stability of the ^66^Ga-label, [^66^Ga]Ga-NOTA-PEG_2_-RM26 was incubated with 1000-fold molar excess of EDTA and PBS for 1 h at room temperature or in human serum for 1 h at 37 °C. Thereafter, ITLC was used to determine the release of ^66^Ga.

NOTA-PEG_2_-RM26 was labeled with ^68^Ga for biodistribution studies by incubating 3 nmol NOTA-PEG_2_-RM26 with 19.2 MBq ^68^Ga-eluate from the ^68^Ge/^68^Ge generator buffered in 1.25 M sodium acetate buffer, pH 3.6. Radiochemical yield was determined by ITLC.

Analytical high performance liquid chromatography (HPLC) was performed (Hitachi Chromaster, with Luna C18 column (5 µm, 100 Å, 150 × 4.6 mm, Phenomenex, Værløse, Denmark) using a gradient of 5% to 70% acetonitrile (with 0.1% TFA) in water over 15 min to study the identity of the radiolabeled products.

### In vitro characterization of [^66^Ga]Ga-NOTA-PEG_2_-RM26

The GRPR-expressing prostate cancer cell line PC-3 was used for all in vitro experiments. Cells were plated one day prior to the experiments. All cell experiments were performed in triplicates.

To test in vitro binding specificity, PC-3 and DU145 (prostate cancer) cells were incubated with 1 nM [^66^Ga]Ga-NOTA-PEG_2_-RM26 for 1 h at 37 °C. GRPR receptors were blocked in half of the samples by pre-incubation with either 200 nM or 600 nM NOTA-PEG_4_-RM26. After incubation, cells were collected using trypsin–EDTA and measured in the automated gamma counter.

To study cellular processing, PC-3 cells were continuously incubated with 2 nM [^66^Ga]Ga-NOTA-PEG_2_-RM26 for up to 24 h at 37 °C. At pre-determined time points (1 h, 2 h, 4 h, 24 h) cells were incubated with 0.2 M glycine buffer (with 0.15 M NaCl, 4 M Urea, pH 2) for 5 min on ice to collect the membrane-bound activity. Thereafter, cells were incubated at 37 °C for 30 min with 1 M NaOH and collected. Activity content in those samples was considered internalized activity.

Binding kinetics of [^66^Ga]Ga-NOTA-PEG_2_-RM26 were measured in real-time using Ligand Tracer (Ridgeview Instruments AB, Uppsala, Sweden). Cells were plated in a dedicated area of 10 cm Petri dish and the measurement was performed according to previously published protocols^49^. In brief, to measure the association, increasing concentrations (in the range 0.5 nM to 10 nM) of [^66^Ga]Ga-NOTA-PEG_2_-RM26 were stepwise added to the cell dish once the cell bound activity of the previous concentration had reached equilibrium. To measure dissociation, the radioactive solution was replaced with fresh cell culture media. Data was treated using TraceDrawer Software (Ridgeview Instruments AB, Uppsala, Sweden) with a 1:1 kinetic binding model.

### Biodistribution and in vivo specificity of [^66^Ga]Ga-NOTA-PEG_2_-RM26

For biodistribution, 40–50 pmol [^66^Ga]Ga-NOTA-PEG_2_-RM26 (40 kBq) per mouse, diluted in 100 µl 1% BSA/PBS, were injected intravenously (iv). Mice were sacrificed 3 h and 22 h pi by intraperitoneal injection of a lethal dose of Ketalar-Rompun solution (10 mg/mL Ketaminol (Intervet) and 1 mg/mL Rompun (Bayer); 20 μL solution/gram of body weight). Tumors were collected as well as samples of blood, lung, liver, spleen, pancreas, stomach, small intestine, kidneys, muscle, and bone. Gastrointestinal (GI) tract and carcass were also collected. Samples were weighed, and measured for activity content in the automated gamma counter. Corrections for background, spill-over and decay were performed for all measurements.

For the in vivo specificity test, mice in the blocking group were co-injected with 10 nmol of non-labeled NOTA-PEG_2_-RM26 and sacrificed 3 h pi and samples were collected according to the protocol described above.

Biodistribution of [^68^Ga]Ga-NOTA-PEG_2_-RM26 3 h pi was included for comparison. Mice were therefore injected with 40–50 pmol (250 kBq) ^68^Ga-NOTA-PEG_2_-RM26, euthanized 3 h pi and samples were collected according to the protocol described above.

### Distribution of ^66^Ga in NMRI mice

^66^Ga-chloride solution was incubated with 1.25 M sodium acetate (pH 3.6) mimicking radiolabeling conditions. The solution was diluted in 1% BSA/PBS and female NRMI mice were injected with 40 kBq ^66^Ga-solution. Three hours pi mice were sacrificed and tissue samples were taken according to the protocol described above.

### Analysis of blood metabolites

NMRI Mice were injected with 1.5 nmol (6.75–7.3 MBq) [^68^Ga]Ga-NOTA-PEG_2_-RM26 and sacrificed 5 min pi by cervical dislocation. Immediately after blood was collected by heart puncture into a pre-chilled heparinized Eppendorf tube. Blood samples were centrifuged (10000RCF, 10 min at 4 °C), plasma was collected and mixed with equal amount of ice cold acetonitrile. The mixture was centrifuged (15,000 RCF, 10 min, 4 °C), the supernatant was collected and sterile filtered with a 0.2 µm PTFE syringe filter. The filtrate was analyzed on HPLC according to the method described above.

### MicroPET/MR imaging GRPR expression in mice using [^66^Ga]Ga-NOTA-PEG_2_-RM26

PC-3 xenograft bearing mice were iv injected with 40 pmol (0.25 MBq) [^66^Ga]Ga-NOTA-PEG_2_-RM26 and microPET/MR imaging was performed 3 h and 22 h pi using a nanoScan PET/MR (Mediso Medical Imaging Systems Ltd., Budapest, Hungary). Mice were under general anesthesia (0.06% sevoflurane; 50%/50% medical oxygen:air) for the 3 h scan and euthanized before the 22 h pi scan. Whole body PET Scans were acquired for 40 min at 3 h pi and 60–140 min at 22 h pi and reconstructed using Tera-Tomo™ 3D reconstruction engine and the matching MR scans for attenuation correction.

MRI was performed immediately after PET acquisition with a 3 T nanoScan PET/MR scanner (Mediso Medical Imaging Systems Ltd., Budapest, Hungary). MRI parameters were as follows:

Whole body MRI scans were performed immediately after PET acquisition with a 3 T nanoScan PET/MR scanner (Mediso Medical Imaging Systems Ltd., Budapest, Hungary) using a T_1_-weighted spin-echo sequence. Parameters were as follows: FOV 80 × 60 mm, acquisition matrix 256 × 192, resolution in plane 0.313 × 0.313 mm, number of accumulations (scans, signal averages) 4, time repetition (TR) 300 ms, echo time (TE) 9 ms, receiver bandwidth (BW) 40,000 Hz.

## Supplementary Information

Supplementary Information.

## References

[1] Körner M, Waser B, Rehmann R, Reubi JC (2014). Early over-expression of GRP receptors in prostatic carcinogenesis. Prostate.

[2] Markwalder R, Reubi JC (1999). Gastrin-releasing peptide receptors in the human prostate: Relation to neoplastic transformation. Cancer Res..

[3] Patel O, Shulkes A, Baldwin GS (2006). Gastrin-releasing peptide and cancer. Biochim. Biophys. Acta.

[4] Reubi, J. C., Wenger, S., Schmuckli-Maurer, J., Schaer, J.-C. & Gugger, M. Bombesin receptor subtypes in human cancers: Detection with the universal radioligand (125)I-[D-TYR(6), beta-ALA(11), PHE(13), NLE(14)] bombesin(6–14). *Clin. Cancer Res. Off. J. Am. Assoc. Cancer Res.* **8**, 1139–1146 (2002). 11948125

[5] Sun B, Halmos G, Schally AV, Wang X, Martinez M (2000). Presence of receptors for bombesin/gastrin-releasing peptide and mRNA for three receptor subtypes in human prostate cancers. Prostate.

[6] Beer M (2012). Profiling gastrin-releasing peptide receptor in prostate tissues: Clinical implications and molecular correlates. Prostate.

[7] Ischia J, Patel O, Bolton D, Shulkes A, Baldwin GS (2014). Expression and function of gastrin-releasing peptide (GRP) in normal and cancerous urological tissues. BJU Int..

[8] Patel O, Dumesny C, Shulkes A, Baldwin GS (2007). C-terminal fragments of the gastrin-releasing peptide precursor stimulate cell proliferation via a novel receptor. Endocrinology.

[9] Levine L (2003). Bombesin stimulates nuclear factor kappa B activation and expression of proangiogenic factors in prostate cancer cells. Cancer Res..

[10] Aprikian AG, Tremblay L, Han K, Chevalier S (1997). Bombesin stimulates the motility of human prostate-carcinoma cells through tyrosine phosphorylation of focal adhesion kinase and of integrin-associated proteins. Int. J. Cancer.

[11] Hoosein Naseema M., Logothetis Christopher J. & Chung Leland W.K. Differential effects of peptide hormones bombesin, vasoactive intestinal polypeptide and somatostatin analog RC-160 on the invasive capacity of human prostatic carcinoma cells. *J. Urol.* **149**, 1209–1213 (1993). 10.1016/s0022-5347(17)36349-8 8097794

[12] Thompson, I. M. *et al.* Prevalence of prostate cancer among men with a prostate-specific antigen level < or = 4.0 ng per milliliter. *N. Engl. J. Med.* **350**, 2239–2246 (2004). 10.1056/NEJMoa031918 15163773

[13] Presti J (2008). Does the yield of prostate cancer biopsy and repeat biopsy justify the frequency of their use?. Nat. Clin. Pract. Urol..

[14] Ananias HJK, van den Heuvel MC, Helfrich W, de Jong IJ (2009). Expression of the gastrin-releasing peptide receptor, the prostate stem cell antigen and the prostate-specific membrane antigen in lymph node and bone metastases of prostate cancer. Prostate.

[15] Baratto L, Jadvar H, Iagaru A (2018). Prostate cancer theranostics targeting gastrin-releasing peptide receptors. Mol. Imaging Biol..

[16] Lantry LE (2006). 177Lu-AMBA: Synthesis and characterization of a selective ^177^Lu-labeled GRP-R agonist for systemic radiotherapy of prostate cancer. J. Nucl. Med..

[17] Mansi R, Minamimoto R, Mäcke H, Iagaru AH (2016). Bombesin-targeted PET of prostate cancer. J. Nucl. Med..

[18] Mitran B, Tolmachev V, Orlova A (2020). Radiolabeled GRPR antagonists for imaging of disseminated prostate cancer. Influence of labeling chemistry on targeting properties. Curr. Med. Chem..

[19] Ginj M (2006). Radiolabeled somatostatin receptor antagonists are preferable to agonists for in vivo peptide receptor targeting of tumors. Proc. Natl. Acad. Sci. U. S. A..

[20] Cescato R (2008). Bombesin receptor antagonists may be preferable to agonists for tumor targeting. J. Nucl. Med. Off. Publ. Soc. Nucl. Med..

[21] Mitran B (2016). Selection of optimal chelator improves the contrast of GRPR imaging using bombesin analogue RM26. Int. J. Oncol..

[22] Mitran B (2017). High contrast PET imaging of GRPR expression in prostate cancer using cobalt-labeled Bombesin antagonist RM26. Contrast Media Mol. Imaging.

[23] Mitran B (2019). Trastuzumab cotreatment improves survival of mice with PC-3 prostate cancer xenografts treated with the GRPR antagonist 177 Lu-DOTAGA-PEG2 -RM26. Int. J. Cancer.

[24] Varasteh Z (2013). Synthesis and characterization of a high-affinity NOTA-conjugated bombesin antagonist for GRPR-targeted tumor imaging. Bioconjug. Chem..

[25] Varasteh Z (2014). The effect of mini-PEG-based spacer length on binding and pharmacokinetic properties of a 68Ga-labeled NOTA-conjugated antagonistic analog of bombesin. Mol. Basel Switz..

[26] Zhang J (2018). PET using a GRPR antagonist 68Ga-RM26 in healthy volunteers and prostate cancer patients. J. Nucl. Med. Off. Publ. Soc. Nucl. Med..

[27] Rahmim A, Zaidi H (2008). PET versus SPECT: Strengths, limitations and challenges. Nucl. Med. Commun..

[28] Nayak TK, Brechbiel MW (2009). Radioimmunoimaging with longer-lived positron-emitting radionuclides: Potentials and challenges. Bioconjug. Chem..

[29] Zweit J, Sharma H, Downey S (1987). Production of gallium-66, a short-lived, positron emitting radionuclide. Int. J. Radiat. Appl. Instrum. (A).

[30] Lewis MR (2002). Production and purification of gallium-66 for preparation of tumor-targeting radiopharmaceuticals. Nucl. Med. Biol..

[31] Graham MC (1997). An investigation of the physical characteristics of 66Ga as an isotope for PET imaging and quantification. Med. Phys..

[32] Rodríguez-Villafuerte, M., Hernández, E. M., Alva-Sánchez, H., Martínez-Dávalos, A. & Ávila-Rodríguez, M. A. Positron range effects of 66Ga in small-animal PET imaging. *Phys. Med. PM Int. J. Devoted Appl. Phys. Med. Biol. Off. J. Ital. Assoc. Biomed. Phys. AIFB* **67**, 50–57 (2019). 10.1016/j.ejmp.2019.10.024 31669670

[33] Engle JW (2012). Positron emission tomography imaging of tumor angiogenesis with a 66Ga-labeled monoclonal antibody. Mol. Pharm..

[34] Lopez-Rodriguez V (2015). Preparation and preclinical evaluation of 66Ga-DOTA-E(c(RGDfK))2 as a potential theranostic radiopharmaceutical. Nucl. Med. Biol..

[35] Amor-Coarasa, A. *et al.* 66Ga: A novelty or a valuable preclinical screening tool for the design of targeted radiopharmaceuticals? *Mol. J. Synth. Chem. Nat. Prod. Chem.* **23** (2018). 10.3390/molecules23102575 PMC6222850 30304795

[36] Ugur Ö (2002). Ga-66 labeled somatostatin analogue DOTA-DPhe1-Tyr3-octreotide as a potential agent for positron emission tomography imaging and receptor mediated internal radiotherapy of somatostatin receptor positive tumors. Nucl. Med. Biol..

[37] Varasteh Z (2015). The effect of macrocyclic chelators on the targeting properties of the 68Ga-labeled gastrin releasing peptide receptor antagonist PEG2-RM26. Nucl. Med. Biol..

[38] Nagasaki S (2012). Immunohistochemical analysis of gastrin-releasing peptide receptor (GRPR) and possible regulation by estrogen receptor βcx in human prostate carcinoma. Neoplasma.

[39] Mitran B (2019). Selection of an optimal macrocyclic chelator improves the imaging of prostate cancer using cobalt-labeled GRPR antagonist RM26. Sci. Rep..

[40] Garousi J (2017). The use of radiocobalt as a label improves imaging of EGFR using DOTA-conjugated affibody molecule. Sci. Rep..

[41] Rinne, S. S. *et al.* Benefit of later-time-point PET imaging of HER3 expression using optimized radiocobalt-labeled affibody molecules. *Int. J. Mol. Sci.* **21** (2020). 10.3390/ijms21061972 PMC7139902 32183096

[42] Heppeler, A. *et al.* Metal-ion-dependent biological properties of a chelator-derived somatostatin analogue for tumour targeting. *Chem. Eur. J.* **14**, 3026–3034 (2008). 10.1002/chem.200701264 18246556

[43] Wadas TJ, Wong EH, Weisman GR, Anderson CJ (2010). Coordinating radiometals of copper, gallium, indium, yttrium and zirconium for PET and SPECT imaging of disease. Chem. Rev..

[44] Heppeler, A. *et al.* Radiometal-labelled macrocyclic chelator-derivatised somatostatin analogue with superb tumour-targeting properties and potential for receptor-mediated internal radiotherapy. *Chem. Eur. J.* **5**, 1974–1981 (1999).

[45] Kaloudi A (2019). Localization of 99mTc-GRP analogs in GRPR-expressing tumors: Effects of peptide length and neprilysin inhibition on biological responses. Pharmaceuticals.

[46] Lymperis E (2019). Comparative evaluation of the new GRPR-antagonist 111In-SB9 and 111In-AMBA in prostate cancer models: Implications of in vivo stability. J. Label. Compd. Radiopharm..

[47] Rodnick M (2019). Clinical implementation of cyclotron-based [68Ga]Ga-PSMA-11 at the University of Michigan. J. Label Compd. Radiopharm..

[48] Nair, M. *et al.* Cyclotron production and automated new 2-column processing of [Ga-68] GaCl_3_. *Eur. J. Nucl. Med. Mol. Imaging* **44**, Suppl 2 (2017).

[49] Björke, H. & Andersson, K. Measuring the affinity of a radioligand with its receptor using a rotating cell dish with in situ reference area. *Appl. Radiat. Isot. Data Instrum. Methods Use Agric. Ind. Med.* **64**, 32–37 (2006). 10.1016/j.apradiso.2005.06.007 16055339

