# Supplementary material for: 66Ga-PET-imaging of GRPR-expression in prostate cancer: production and characterization of [66Ga]Ga-NOTA-PEG2-RM26
Source: Sci Rep. 2021 Feb 11;11:3631. doi: 10.1038/s41598-021-82995-7 (PMC7878787; doi:10.1038/s41598-021-82995-7)
Supplement: Supplementary file 1 — Supplementary Information. [file 41598_2021_82995_MOESM1_ESM.docx]

Supplementary Material

**^66^Ga-PET-Imaging of GRPR-expression in prostate cancer: Production and characterization of [^66^Ga]Ga-NOTA-PEG_2_-RM26**

Sara S. Rinne^1^, Ayman Abouzayed^1^, Katherine Gagnon^2^, Vladimir Tolmachev^3,4^, Anna Orlova^1,4,5^

**Results**

**Table S1: In vitro stability of [^66^Ga]Ga-NOTA-PEG_2_-RM26.** [^66^Ga]Ga-NOTA-PEG_2_-RM26 was incubated for one hour in presence of PBS, 1000x molar excess of EDTA at room temperature or human serum at 37 °C. the release of ^66^Ga was determined by ITLC.

|  | **% Release of ^66^Ga** |
| --- | --- |
| PBS | 0.4 ± 0.1 |
| 1000x molar excess of EDTA | 0.6 ± 0.8 |
| Human serum | 0.17 ± 0.06 |

**Figure S1. Gamma-spectrum of ^66^Ga.**

**Figure S2. Decay of ^66^Ga presented as a standard (A) or semi-logathimic (B) plot.**

**
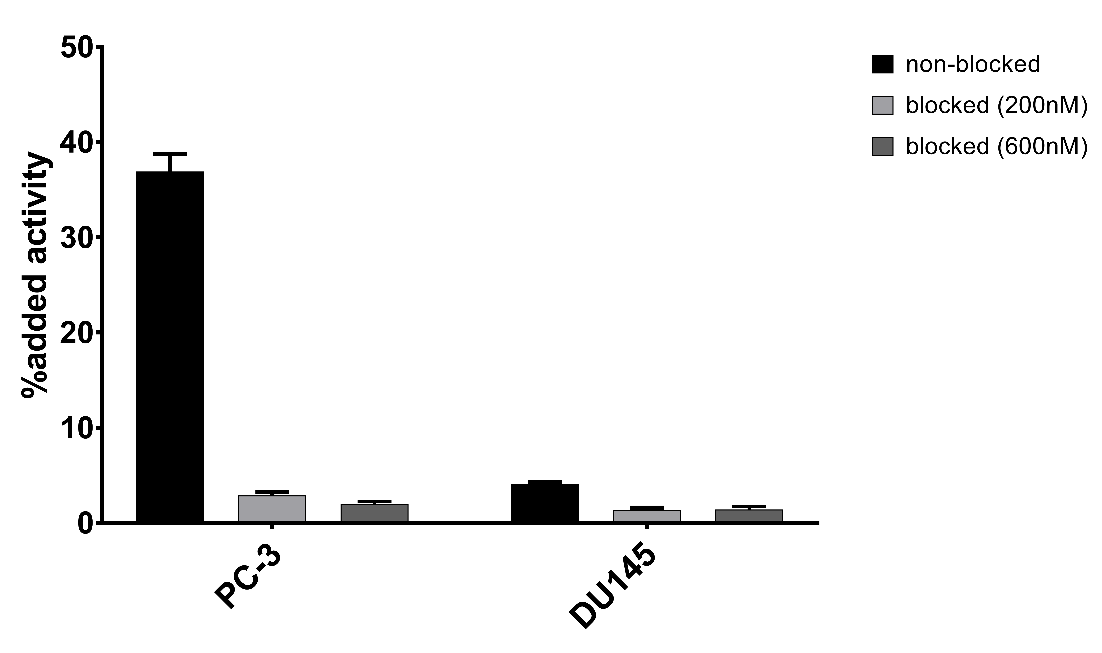
**

**Figure S3: In vitro specificity of [^66^Ga]Ga-NOTA-PEG_2_-RM26 in GRPR expressing PC-3 and DU145 prostate cancer cells.** Before addition of the radiolabeled peptide, receptors in the blocked groups were saturated by addition of NOTA-PEG_4_-RM26.

**Figure S4: Distribution of ^66^Ga-Chloride in NMRI mice 3 h pi.** Mice were injected with 40 kBq ^66^Ga-Chloride solution diluted in 1% BSA/PBS. Prior to injection, ^66^Ga-Chloride was incubated with 1.25 M sodium acetate buffer (pH 3.6) for 15 min at 85°C prior to injection to mimic radiolabeling


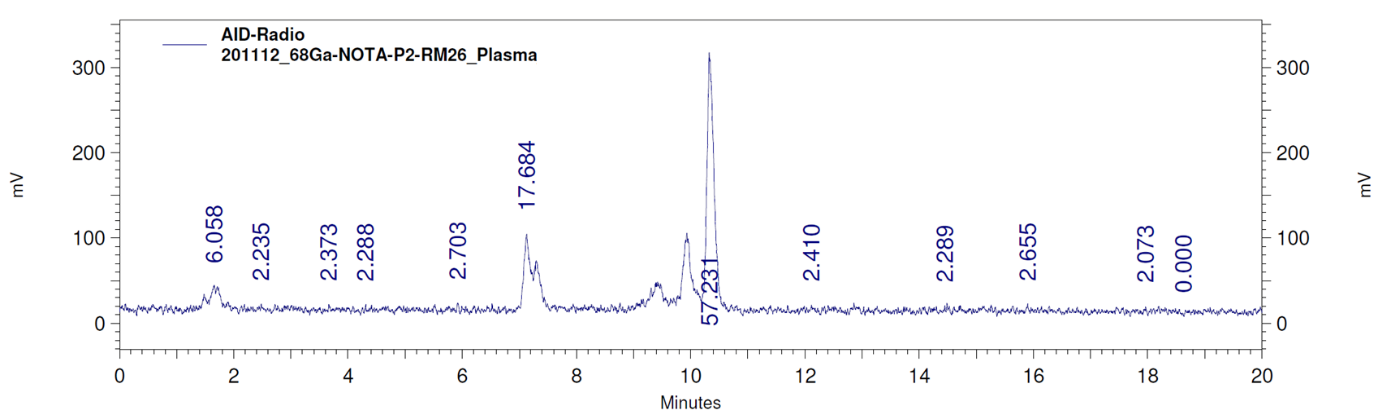


**Figure S5: Representative radio-HPLC chromatogram of blood metabolites** of [ ^68^Ga]Ga-NOTA-PEG_2_-RM26 5 min pi. NMRI mice were injected with 6.75 – 7.3 MBq (1.5 nmol) [ ^68^Ga]Ga-NOTA-PEG_2_-RM26.
